# Eating disorders during lockdown: the transcultural influence on eating and mood disturbances in Ibero-Brazilian population

**DOI:** 10.1186/s40337-023-00762-7

**Published:** 2023-03-11

**Authors:** Isabel Baenas, Carmem Beatriz Neufeld, Rita Ramos, Lucero Munguía, Rosane P. Pessa, Tânia Rodrigues, Susana Jiménez-Murcia, Sónia Gonçalves, Marília C. Teodoro, Ana Pinto-Bastos, Nazaré O. Almeida, Roser Granero, Mikel Etxandi, Shauana R. S. Soares, Fernando Fernández-Aranda, Paulo P. P. Machado

**Affiliations:** 1 Eating Disorders Unit, Department of Psychology, Bellvitge University Hospital-IDIBELL, 08907 Barcelona, Spain; 2 CIBER Physiopathology of Obesity and Nutrition (CIBERobn), Instituto de Salud Carlos III, 28029 Barcelona, Spain; 3 Psychoneurobiology of Eating and Addictive Behaviors Group, Neurosciences Programme, Bellvitge Biomedical Research Institute (IDIBELL), 08908 Barcelona, Spain; 4 Department of Psychology, Faculty of Philosophy, Sciences and Languages of Ribeirão Preto, University of São Paulo, São Paulo, Brazil; 5 Psychotherapy and Psychopathology Lab—Psychology Research Center, School of Psychology, University of Minho, 4710-057 Braga, Portugal; 6 Department of Maternal-Infant and Public Health Nursing, University of São Paulo at Ribeirão Preto College of Nursing, São Paulo, Brazil; 7 Department of Clinical Sciences, School of Medicine and Health Sciences, University of Barcelona, 08907 Barcelona, Spain; 8 Department of Psychobiology and Methodology, Autonomous University of Barcelona, 08193 Barcelona, Spain; 9 Department of Psychiatry, Hospital Universitari Germans Trias I Pujol, IGTP Campus Can Ruti, Badalona, Spain; 10 Program Public Health Nursing, University of São Paulo at Ribeirão Preto College of Nursing, Ribeirão Preto, Brazil

**Keywords:** Eating disorders, Lockdown, COVID-19 isolation eating scale (CIES), Mood disturbances, Transcultural study

## Abstract

**Background:**

COVID-19 pandemic has implied exceptional restrictive measures to contain its widespread, with adverse consequences on mental health, especially for those people with a background of mental illness, such as eating disorders (EDs). In this population, the influence of socio-cultural aspects on mental health has been still underexplored. Then, the main aim of this study was to assess changes in eating and general psychopathology in people with EDs during lockdown regarding the ED subtype, age, and provenance, and considering socio-cultural aspects (e.g., socioeconomical factors such as work and financial losses, social support, restrictive measures, or health accessibility, among others).

**Methods:**

The clinical sample was composed of 264 female participants with EDs (74 anorexia nervosa (AN), 44 bulimia nervosa (BN), 81 binge eating disorder (BED), and 65 other specified feeding and eating disorder (OSFED)), with a mean age of 33.49 years old (*SD* = 12.54), from specialized ED units in Brazil, Portugal, and Spain. The participants were evaluated using the COVID-19 Isolation Eating Scale (CIES).

**Results:**

A global impairment in mood symptoms and emotion regulation was reported in all the ED subtypes, groups of age, and countries. Spanish and Portuguese individuals seemed more resilient than Brazilian ones (p < .05), who reported a more adverse socio-cultural context (i.e., physical health, socio-familial, occupational, and economic status) (p < .001). A global trend to eating symptoms worsening during lockdown was observed, regardless of the ED subtype, group of age, and country, but without reaching statistical significance. However, the AN and BED groups described the highest worsening of the eating habits during lockdown. Moreover, individuals with BED significantly increased their weight and body mass index, similarly to BN, and in contrast to the AN and OSFED groups. Finally, we failed to find significant differences between groups of age although the younger group described a significant worsening of the eating symptoms during lockdown.

**Conclusions:**

This study reports a psychopathological impairment in patients with EDs during lockdown, being socio-cultural aspects potential modulatory factors. Individualized approaches to detect special vulnerable groups and long-term follow-ups are still needed.

**Supplementary Information:**

The online version contains supplementary material available at 10.1186/s40337-023-00762-7.

## Abstract (highlights)

Adverse consequences on mental health derived from COVID-19 pandemic and lockdown particularly affected people with mental illness, including eating disorders (EDs), being the involvement of socio-cultural features poorly studied. We aimed to assess eating and mood changes in EDs during lockdown regarding ED subtypes, age, provenance, and considering socio-cultural aspects. 264 females with EDs linked to specialized ED units in Brazil, Portugal, and Spain were evaluated using the COVID-19 Isolation Eating Scale (CIES). A global impairment in mood symptoms and emotion regulation was reported regardless ED subtype, age, and country. Spanish and Portuguese individuals seemed more resilient than Brazilian ones, who reported a more adverse socio-cultural context. A global trend to eating symptoms worsening was observed, regardless of the ED subtype, age, and country, but without statistical significance. Patients with anorexia nervosa and binge eating disorder (BED) described eating style worsening. Moreover, the BED group significantly increased weight and body mass index, similarly to bulimia nervosa, and in contrast to the other subtypes. In sum, a psychopathological impairment was observed in EDs during lockdown, suggesting that socio-cultural aspects could be considered as potential modulatory factors. Nevertheless, these are preliminary results, being longitudinal studies and long-term follow-ups still needed. This work also highlights the importance of more personalized therapeutic approaches.

**Supplementary Information:**

The online version contains supplementary material available at 10.1186/s40337-023-00762-7.

## Introduction

From the end of 2019, the widespread of COVID-19 infection has categorized it as a pandemic [1]. The implementation of different restrictive measures has limited mobility and favored social isolation at home, with a global impact on individuals’ lifestyle on both physical and mental health [2]. In this line, different authors have reported a predominant negative impact of confinement on eating patterns, physical activity, and emotional well-being in general population, including countries from the same geographic area (i.e., Spain and Portugal) or with shared socio-cultural aspects, such as speaking language and historical background (i.e., Portugal and Brazil) [2–4].

Lockdown in Portugal was instituted between 19th March and 3rd May 2020 [5], similarly to Spain, where the state of alarm was decreed from 14th March to 21th June (https://www.boe.es/eli/es/rd/2020/03/14/463). Brazil did not establish neither an official lockdown nor national restrictive measures, which were unequally applied by local governors. As a result, Brazil was considered in the top five countries with a high number of contagious people (https://www.worldometers.info/coronavirus/). Even to date, high differences are found between the three countries. Brazil has 34,470,776 of total cases, and 684,369 deaths, while Spain has a total of 13,352,019 cases and 112,804 deaths, and Portugal 5,429,340 cases and 24,886 deaths (https://covid19.who.int/). Then, this differential lockdown context may influence the impact of the pandemic on the population in each country, together with other idiosyncratic aspects such as the amount and structure of the population, the economic situation of each country, the type of health system, and accessibility to assistance, among others, contributing to perceived emotional distress [2].

Worldwide, people with a background of mental illness were considered as an especially vulnerable group during the pandemic and lockdown [6], including individuals with eating disorders (EDs) [7]. Particularly among them, changes in eating psychopathology and psychological state have been reported [7, 8], being greater than in healthy controls [9], with a tendency to worsen eating symptoms [10].

In Portuguese clinical population, Machado et al. [11], evaluated the impact of the lockdown in 43 adult individuals (95.3% females) with EDs through a self-reported survey: 20 with anorexia nervosa (AN), 14 with bulimia nervosa (BN), two with binge eating disorder (BED), and seven with other specified feeding and eating disorders (OSFED). Notably, 31% of the sample reported an increased weight during lockdown. Besides, a significant increase of body mass index (BMI) in the total sample was stated after lockdown. Most participants, whether in treatment or not during lockdown, described significant changes in their lifestyle, including physical exercise and eating habits, as well as stress linked to the pandemic situation. A higher impact of lockdown was significantly associated with the presence of eating and general psychopathology, but also with impulsivity and difficulties in emotion regulation (ER). Indeed, the ER difficulties mediated the impact of lockdown on the global clinical impairment among these patients. In this vein, emotion dysregulation and a lack of adaptive coping strategies, together with some personality traits (e.g., low self-directedness) have been considered as vulnerability factors leading to psychological distress during lockdown, which might also be associated with disturbed eating patterns not only in the general population [3], but also in individuals with EDs [12, 13].

Previous literature in Spanish clinical population has suggested that the effects of lockdown were different depending on the ED subtypes [14], being individuals with OSFED those who reported the highest global impairment in eating psychopathology [14]. There are few Brazilian studies that evaluated eating patterns during lockdown, without referring to the clinical population with EDs. In the non-clinical population, we found some interesting results. For instance, an increase of the consumption of bakery and processed foods was also accompanied by an inverted eating pattern, with a decrease in food consumption in the morning and an increase at night [15]. Another study that considered adults with Diabetes Type I and II reported that 75.8% of the individuals had eating psychopathology in the context of lockdown [16].

Overall, these results were consistent with research hypotheses that pointed out that the special circumstances of the lockdown may have favored not only an eating style worsening, but also have contributed to exacerbate unhealthy eating patterns among patients with EDs, such as overeating, or even precipitate an ED onset [17]. In this regard, younger age has been highlighted as a possible vulnerability factor related to the negative impact on mental health due to pandemic and lockdown [18] although this aspect was underexplored among people who already had an ED [19]. Moreover, the lockdown and restrictive measures have involved changes in treatment approaches, including the closure of day hospitals and outpatient facilities, or the adaptation to telehealth, which may also have contributed to a higher emotional distress (e.g., anxiety, mood disturbances) [7] and, therefore, to a negative effect in eating symptoms [11].

Early research in individuals with EDs has shown a worsening of their psychological state with anxiety, stress, and increased worries about the risk of being infected with COVID-19 and other negative consequences of the lockdown (e.g., relatives’ infection, employment) [7, 12]. Curiously, mixed differences were found according to the ED subtype. Some studies stated that patients with AN had experienced the highest psychological distress [20]. Other works found that again individuals with OSFED reported the highest rates of anxiety and depressive symptoms after lockdown [14], reinforcing the idea that a worse psychological state could influence eating symptoms [14].

Considering that the heterogeneity of previous results may be influenced by the lack of homogenized psychometric instruments to evaluate changes in eating and mood psychopathology in the context of pandemic and lockdown, the COVID Isolation Eating Scale (CIES) was developed by the *Psychoneurobiology of Eating and Addictive Disorders* Spanish research group [14]. The scale has been validated and translated into nineteen languages [14]. An international and multicentric group of experts from different ED units used it to measure eating and general psychopathological changes during confinement in individuals with EDs [21]. The authors pointed out that while individuals with OSFED indeed were those who reported a worse psychological state during lockdown, the highest impact on weight and eating symptoms was associated with BED, in comparison with other EDs. Interestingly, differences according to cultural context and age were also reported, concluding that Asian and younger individuals appeared to be more resilient than European and adults with EDs, respectively [21]. However, no clinical groups from South America were included in the study.

To the best of our knowledge, this is the first work aimed to explore eating and mood state during lockdown in a clinical sample with EDs from the Ibero-Brazilian community regarding ED subtype, age, provenance, and the socio-cultural context. On the one hand, we analyzed whether there were intra-group pre-post changes in eating and general psychopathology within each ED subtype, group of age, and country. On the other hand, we performed between-group comparisons, according to ED subtypes, groups of age, and provenance. In this study, we used a validated instrument such as CIES, which allowed us to assess eating symptoms and style, anxiety, and depressive symptoms, as well as ER strategies. Moreover, we explored the socio-cultural context (e.g., work status and financial losses, social support, restrictive measures, health accessibility, telehealth implementation, among others), as some of these features have been recognized as potential contributing factors of emotional distress and a worse psychological state [22–24]. Bearing in mind results from previous literature, we hypothesized a global intra-group trend to worsening of eating and mood symptoms in the context of lockdown. Going one step further, we suggested the presence of significant differences in eating and mood changes in between-groups comparisons, with a particular influence of the socio-cultural features in the contextualization of the differences between countries.

## Materials and methods

### Participants

This cross-sectional study was composed by a sample of *N* = 264 participants with a mean age of 33.49 years old (*SD* = 12.54) from private and public ED units in Brazil (*n* = 101), Portugal (*n* = 28) and Spain (*n* = 135). Age range in the study was between 14 and 70 years old. In the study, two groups of age were considered: adolescents and young adulthood (younger than 25 years-old) versus adulthood (25 years old and older). The choice of this cut off point is based in most psychological studies that consider that adolescence now runs up until the age of 25 for the aims of analyzing and treating young people behaviors [25]. As inclusion criteria, all the participants were females diagnosed with an ED, according to Diagnostic and Statistical Manual of Mental Disorders, fifth edition, (DSM-5) criteria [26], by expert clinical psychologists and psychiatrists. All the participants included in this study fully completed the assessments.

### Contextual information

The Iberian countries are integrated by Spain and Portugal. Lockdown in Portugal was instituted between 19th March and 3rd May 2020 [5] and between 14th March and 11th May 2020 in Spain (although the state of alarm was extended until 21th June). In Brazil, around the 27th March 2020 some of the local state governors imposed quarantine although more precise data on the duration of this period is not available.

### Assessment

The COVID Isolation Eating Scale (CIES) is a self-report questionnaire that evaluates the impact of confinement on patients with EDs [14]. It is composed by four subscales: I, referred to COVID-19 pandemic personal circumstances (8 items); II, related to eating psychopathology during confinement (13 items), together with the presence of other psychiatric comorbidities and diabetes mellitus; III (34 items), regarding eating style, general psychopathology, and ER; and IV (13 items), associated with the evaluation of telemedicine. The last three subscales are answered in a five-point Likert scale, and subscales II and III are referred to two moments, before and after lockdown [14]. After a factorial analysis (CFA), five factors were identified [14]. Factor 1 (F1) was defined by the items measuring eating-related symptoms (subscale II); Factor 2 (F2), by the items measuring the effects of lockdown on the eating-related style (subscale III); Factor 3 (F3), by the items assessing anxiety and depressive symptoms (subscale III); Factor 4 (F4) was defined by the items related to ER (subscale III); and Factor 5 (F5), by those that evaluate telemedicine (subscale IV). In this study, F5 (subscale IV) was not evaluated.

Other socio-cultural and contextual information was also collected (e.g., age, work status, economic problems, social support, and health state) (see Additional File 1).

### Procedure

All the participants were already involved in outpatient treatment modality in specialized units of the different countries. Data collection took place retrospectively between June 2020 and March 2021. The subjects were asked by therapists from each centre to voluntarily participate, completing once the required information in reference to the first/early lockdown: some subscales within the CIES Scale, as well as additional data regarding socio-cultural and contextual lockdown were asked regarding two moments, before and after lockdown.

### Statistical analysis

Stata17 for Windows was used for the statistical analysis [27]. The post–pre differences/changes were generated for the weight (kg), the body mass index (BMI) (kg/m^2^), and the CIES factor scores (the absence of changes comparing the post- versus the pre-measures provided a difference equal to zero, positive differences indicated a decreasing trend, and negative differences indicated an increasing trend). Repeated measures analysis of variance (repeated-ANOVA) tested the significance relevance for the differences, and it was implemented through the *manova* command in Stata, which allows fitting mixed designs including controlled variables. The diagnostic subtype, age and, country were included as covariates in the study. Standardized Cohen’s-*d* coefficients measured the effect size for the differences between the means (null effect size was considered for |*d*|< 0.20, low-poor for |*d*|> 0.20, moderate-medium for |*d*|> 0.50 and large-high for |*d*|> 0.80) [28, 29]. The Finner’s method (family-wise error rate -FWER- algorithm more powerful than the classical Bonferroni’s correction) was employed for controlling the increase in the Type-I error due the use of multiple significance tests [30].

## Results

### Characteristics of the participants

Most participants in the study lived with other people during the lockdown (only 29 individuals reported living alone, 11.0%), were not infected by COVID-19 (86%), did not have infected relatives or other close people (57.2%), did not have the responsibility of caring for infected relatives (64.8%), were active at work (52.7%), and did not report economic difficulties in the context of the confinement (59.5%). Additional file 1 displays the distribution of the age and the contextual variables registered during the lockdown (see Additional file 1).

### Intra- and between-group comparisons regarding diagnostic subtypes

Table 1 contains the post–pre changes in the weight (kg), the BMI (kg/m^2^) and CIES subscales scores within each diagnostic subtype. The repeated measures ANOVA adjusted by age and country showed increase for the CIES F3 anxiety/depression and F4 emotion dysregulation among all the ED subtypes. Additionally, patients with BN also increased the CIES F1 eating-related symptoms and F2 eating-related style; BED patients increased weight and BMI mean values.

**Table 1:** Assessment of the post–pre changes stratified by ED-subtype

	Anorexia Nervosa (n = 74)						Bulimia (n = 44)					
	Pre		Post				Pre		Post			
	Mean	SD	Mean	SD	p	|d|	Mean	SD	Mean	SD	p	|d|
Weight (kg)	48.85	9.75	47.08	8.82	.061	0.19	62.96	12.10	63.94	13.03	.244	0.08
BMI (kg/m^2^)	18.84	3.26	18.28	3.99	.141	0.15	23.81	5.32	24.17	5.66	.252	0.07
CIES-F1 ED symptoms	15.07	6.06	15.81	5.90	.195	0.12	18.55	5.52	20.46	6.65	.041*	0.31
CIES-F2 Eating style	13.08	8.86	12.58	8.32	.398	0.06	20.64	9.67	23.68	11.19	.012*	0.29
CIES-F3 Anxiety-depress	19.66	8.95	24.77	9.13	< .001*	**0.56**^**†**^	19.39	10.07	24.64	11.63	< .001*	**0.51**^**†**^
CIES-F4 Emotion dysreg	9.16	4.35	10.57	4.86	< .001*	0.30	9.52	4.95	11.02	5.57	.001*	0.28

	BED (n = 81)						OSFED (n = 65)					
	Pre		Post				Pre		Post			
	Mean	SD	Mean	SD	p	|d|	Mean	SD	Mean	SD	p	|d|
Weight (kg)	85.59	19.58	89.29	21.36	< .001*	0.18	72.15	19.47	71.97	22.01	.868	0.01
BMI (kg/m^2^)	32.30	6.66	33.70	7.37	< .001*	0.20	27.04	7.40	26.95	8.17	.821	0.01
CIES-F1 ED symptoms	16.46	4.03	17.07	4.19	.209	0.15	15.66	5.40	15.89	5.39	.735	0.04
CIES-F2 Eating style	26.70	8.76	26.65	9.19	.960	0.01	17.62	9.00	19.06	9.63	.151	0.16
CIES-F3 Anxiety-depress	18.83	7.74	24.82	8.54	< .001*	**0.73**^**†**^	18.22	8.00	23.98	8.47	< .001*	**0.70**^**†**^
CIES-F4 Emotional dysreg	8.44	4.30	9.67	4.41	< .001*	0.28	7.86	3.91	9.26	3.91	.008*	0.36

ED: eating disorder. BMI: body mass index. BED: binge eating disorder. OSFED: other specificized feeding and eating disorders. Emotion dysreg.: emotional dysregulation. SD: standard deviation. *Bold: significant comparison. ^†^Bold: Effect size into the ranges moderate to large. Results adjusted by age and country

**Fig. 1:**
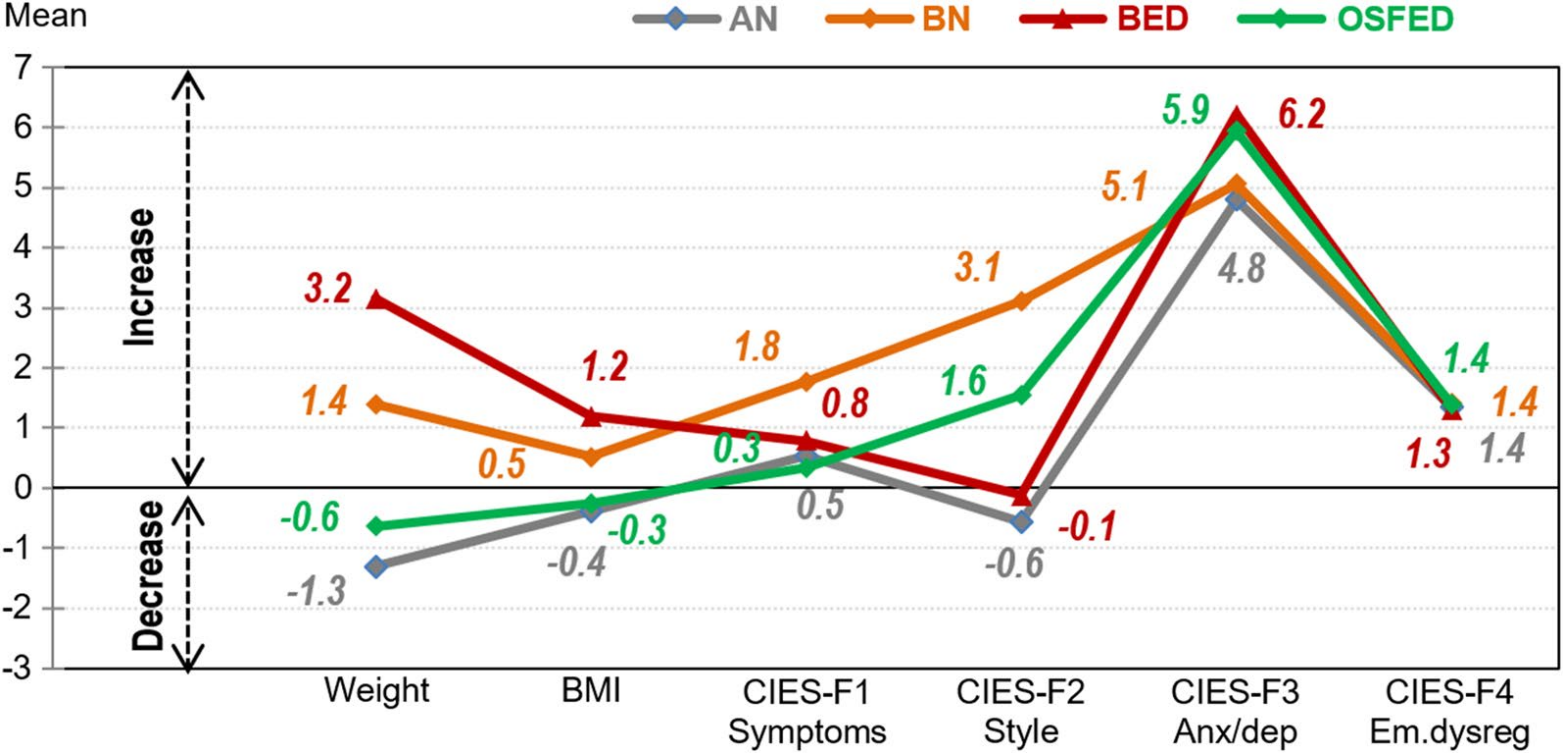
Mean post–pre changes by country. *Note.* BMI: body mass index. AN: anorexia nervosa. BN: bulimia nervosa. BED: binge eating disorder. OSFED: other specified feeding eating disorder

Figure 1 shows the mean scores for the post–pre changes between groups (defined as the difference between the measures at the end of the lockdown versus the measures prior to the lockdown) (see Additional file 2). After the adjustment by age and country, the mean changes for weight and BMI were statistically equal comparing AN versus OSFED (both groups decreased) and comparing BN versus BED (both groups increased). For the CIES F2 eating-related style, BN and OSFED achieved similar mean increase, while a brief decrease was reported for AN and BED.

### Intra- and between-group comparisons regarding groups of age

The results of the repeated ANOVA exploring the changes between the post- and pre-lockdown within each age group (young/adolescents and adults) are displayed in Table 2. After the adjustment by the ED subtype and country, it was observed a significant increase in the CIES F1 eating-related symptoms and CIES F3 anxiety/depression scales among the young/adolescents subsample. For the adult subsample, a significant increase was observed in the weight, the BMI, the CIES F3 anxiety/depression, and the CIES F4 emotional dysregulation.

**Table 2:** Assessment of the post–pre changes by groups of age

	Young/adolescents (n = 78)						Adults (n = 186)					
	Pre		Post				Pre		Post			
	Mean	SD	Mean	SD	p	|d|	Mean	SD	Mean	SD	p	|d|
Weight (kg)	63.40	15.13	62.64	16.21	.451	0.05	67.72	22.20	69.08	24.39	.044*	0.06
BMI (kg/m^2^)	24.39	6.02	24.15	6.63	.513	0.04	25.71	7.95	26.27	8.81	.035*	0.07
CIES-F1 ED symptoms	15.60	5.51	17.45	6.46	.030*	0.31	16.98	5.20	17.44	5.29	.228	0.09
CIES-F2 Eating style	17.40	9.47	17.79	9.91	.679	0.04	20.59	10.17	21.51	10.67	.141	0.09
CIES-F3 Anxiety-depress	18.28	9.12	24.18	9.44	.001*	**0.64**^**†**^	19.72	8.28	24.52	9.15	.001*	**0.55**^**†**^
CIES-F4 Emotional dysreg	9.19	4.68	10.26	4.90	.065	0.22	8.73	4.21	10.00	4.55	.001*	0.29

ED: eating disorder. BMI: body mass index. Emotion dysreg.: emotional dysregulation. SD: standard deviation. *Bold: significant comparison. ^†^Bold: Effect size into the ranges moderate to large. Results adjusted by ED-subtype and country

**Fig. 2:**
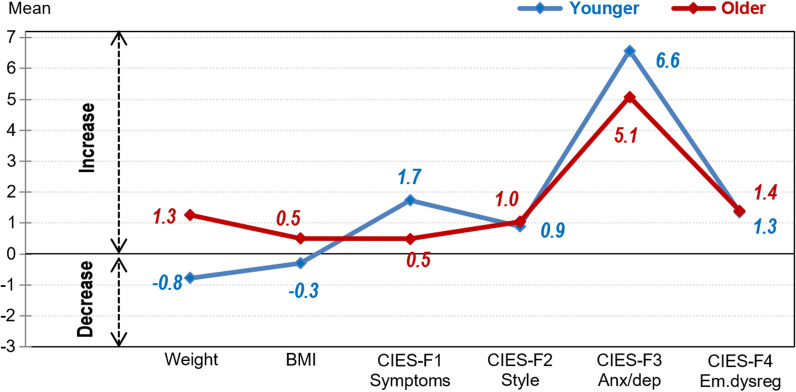
Mean post–pre changes by age.* Note*: BMI, body mass index

The results of the ANOVA procedures comparing the post–pre changes during the lockdown between the groups of age (also adjusted by the ED subtype and the country) showed no differences (see Fig. 2 and Additional file 3).

### Intra- and between-group comparisons regarding country

The results of the repeated-ANOVA stratified by country and adjusted by the ED-subtype and age are shown in Table 3. For the three countries, a significant increase was identified in the CIES F3 anxiety-depression scale. In addition, an increase in the CIES F4 emotion dysregulation scale was observed for patients living in Brazil and Spain.

**Table 3:** Assessment of the post–pre changes stratified by country

Portugal *(n* = *28)*	Pre		Post			
	*Mean*	*SD*	*Mean*	*SD*	*p*	*|d|*
Weight (kg)	58.75	17.56	59.58	20.23	.410	0.04
BMI (kg/m^2^)	22.67	7.38	23.00	8.36	.398	0.04
CIES-F1 ED symptoms	16.69	5.37	17.41	7.91	.499	0.11
CIES-F2 Eating style	18.48	9.54	18.57	9.20	.939	0.01
CIES-F3 Anxiety-depression symptoms	21.84	7.75	24.01	8.32	.043*	0.27
CIES-F4 Emotional dysregulation	9.21	4.35	9.45	4.32	.527	0.06

Brazil (n = 101)	Pre		Post			
	Mean	SD	Mean	SD	p	|d|
Weight (kg)	67.42	17.75	68.75	20.70	.316	0.07
BMI (kg/m^2^)	25.58	6.37	26.12	7.51	.273	0.08
CIES-F1 ED symptoms	17.40	4.59	18.54	4.77	.113	0.24
CIES-F2 Eating style	24.42	7.63	25.89	8.69	.226	0.18
CIES-F3 Anxiety-depression symptoms	19.27	7.29	27.08	8.26	< .001*	**1.00**^**†**^
CIES-F4 Emotional dysregulation	8.76	3.80	10.64	4.19	< .001*	0.47

Spain (n = 135)	Pre		Post			
	Mean	SD	Mean	SD	p	|d|
Weight (kg)	70.39	23.34	70.29	24.88	.879	0.00
BMI (kg/m^2^)	26.50	8.44	26.47	9.12	.921	0.00
CIES-F1 ED symptoms	15.93	5.83	16.61	5.72	.153	0.12
CIES-F2 Eating style	16.53	10.35	16.76	10.24	.701	0.02
CIES-F3 Anxiety-depression symptoms	18.95	9.50	23.88	10.01	< .001*	**0.50**^**†**^
CIES-F4 Emotional dysregulation	8.88	4.75	10.20	5.05	< .001*	0.27

ED: eating disorder. BMI: body mass index. SD: standard deviation. *Bold: significant comparison. ^†^Bold: Effect size into the ranges moderate to large. Results adjusted by ED-subtype and age.

**Fig. 3:**
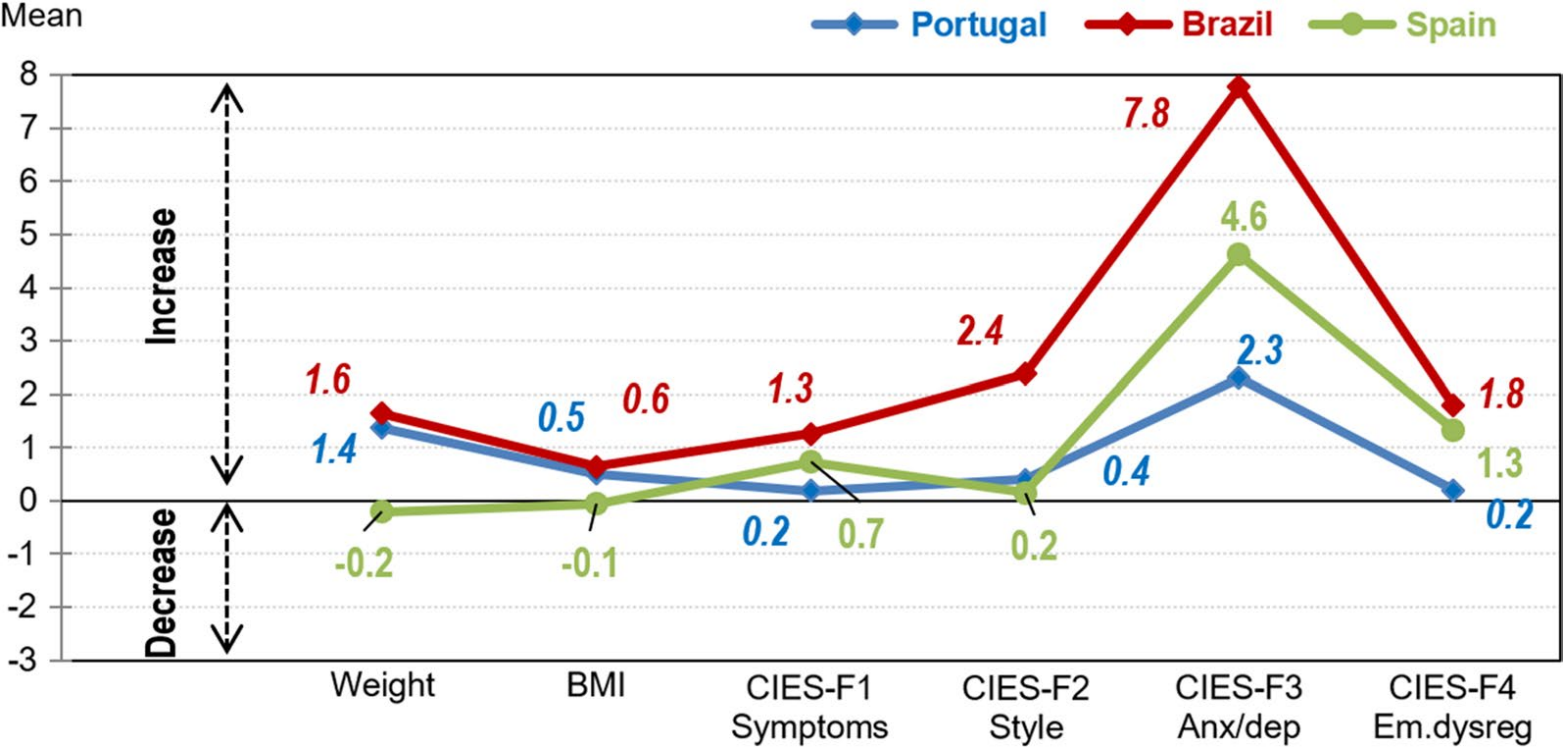
Mean post–pre changes by country.* Note*: BMI, body mass index

The comparison of the post–pre changes between the countries (adjusted by the ED-subtype and age) is contained in Fig. 3 and Additional file 4. Patients living in Brazil reported higher positive changes in the CIES scales (except for the F1 eating-related symptom levels).

## Discussion

The main aim of this work was to evaluate the impact of COVID-19 lockdown in eating symptomatology and general psychopathology in patients with EDs from Ibero-Brazilian countries, considering the CIES Scale, as well as other socio-cultural and contextual factors. We also performed between-groups comparisons regarding ED subtypes, age, and provenance.

A global trend towards the impairment of eating symptoms pre to post lockdown was described in all ED subtypes [7, 11], reaching statistical significance in the BN group. The last result contrasted with previous studies that described an absence of pre-post changes [14], or even a decrease in eating symptoms, among individuals with BN during lockdown [21]. In our study, surprisingly, these patients also reported a significant improvement in their eating style, similarly to the OSFED group. Likewise, changes in eating style were more alike between individuals with AN and BED, who reported eating style worsening. This result partially agreed with previous literature which referred to BED and BN as the ED subtypes with the highest negative impact on eating style [21]. In our case, a higher trend to control food intake and restriction in patients with AN [8, 9] whereas disordered eating such as grazing eating behavior among individuals with BED may support these findings [7].

Remarkably, the AN and OSFED group had significant differences in weight and BMI changes in comparison with the BED and BN groups, which also appeared to be more similar to each other. While a trend towards weight loss, with a consequent reduction in BMI, was observed in individuals with AN and OSFED, the opposite occurred in subjects with BED and BN [21, 31]. In fact, intra-group comparisons showed that the BED group reported a significant increase in weight and BMI after lockdown [21]. Restrictive measures, sedentarism, and food insecurity may be contributing factors [32]. However, our results revealed that neither of the two weight approaches were significantly superior to the other. Thus, a balanced weight change between ED subtypes may be deduced in contrast with previous literature [21].

Despite both groups of age (i.e., adolescent/young and adults) reported worse eating symptoms after lockdown, in the case of younger patients this change was statistically significant. Works related to young people with a diagnostic of ED showed a psychopathological impairment in the context of pandemic characterized by a higher difficulty in achieving goal weight, as well as a higher hospitalization rate [33]. Lower food security, changes in academic routines, and stress due to pandemic with affective implications have been pointed as some of the factors potentially involved [34]. However, we failed to find significant pre-post differences between adolescents/young individuals with EDs and adult patients neither in weight/BMI changes, eating symptoms, nor in psychological state. In the general population, younger age has been proposed as a contributing factor for suffering from a more negative impact on mental health during pandemic [18], including disordered eating [33, 34] and the development of an ED [35]. On the other hand, our results also contrast with the hypothesis of a higher resilience among younger patients in comparison with adults regarding people with EDs [21]. The global tendency to an impairment of the eating and mood symptoms in both groups during lockdown could partially contribute to explain our findings, in line with Monteleone et al. [19]. Indeed, an increased need for ED assistance has been described in the context of the pandemic in both adolescents/young and adults individuals with EDs [36].

While the younger group described a mild non-significant loss of weight, the adult group experienced a significant increase of weight and BMI during lockdown. Although our results have been adjusted by ED subtype, patients with younger age would presumably have a higher prevalence of AN and OSFED diagnosis than BED, more frequent among adult individuals [37]. Moreover, both groups showed an eating style worsening. In this regard, we hypothesize that the impaired eating pattern in younger patients could be more linked to restrictive behaviors and exercise practice, in line with the observed trend to control weight. On the other hand, changes in eating style among adults might be closely associated with increased food consumption (e.g., picking, binging), which, consequently, could be more probably linked with a weight increase.

To the best of our knowledge, this is the first study which includes European countries (Iberian countries, Spain and Portugal) with South American ones (i.e., Brazil). Previous studies analyzing eating and mood psychopathology between different continents are scarce without grouping European and South American countries [21]. Curiously, in the later work, authors reported that Asian patients seemed more resilient than European individuals with EDs, who reported worse eating symptomatology during the lockdown [21]. Using the same instrument (i.e., CIES), the present study did not find significant differences related to eating psychopathology, nor weight changes between European and South American individuals, regardless of the ED subtype and age. Indeed, we observed that the three groups of provenance reported a trend towards worsening of their eating symptoms. Despite these results would be in line with the global impairment of eating symptoms described among patients with EDs in the context of the lockdown [7, 8, 10, 11], it also highlights the need to design future studies that include large international samples to contrast whether the impact of the pandemic and lockdown on eating symptoms in individuals with EDs would be more similar between some continents, as well as which kind of socio-cultural and contextual features could be modulating this fact.

Interestingly, after lockdown, the self-reported anxiety and depressive symptoms evaluated with the CIES scale were higher and statistically significant than in the pre-lockdown in all the ED subtypes, age groups, and countries, and generally accompanied by an impaired ER [11, 19]. Even that a concern for patients with EDs was expressed from the beginning of the COVID-19 pandemic [7], current studies have supported that this population has been highly impacted by this health crisis [38, 39]. Going one step further, as a result of the between-groups comparisons, only the comparison between countries showed significant differences. Curiously, the Brazilian group described a worse psychological state in the context of the lockdown when compared with Iberian countries. In this line, a previous study found higher anxiety in the Brazilian population than in the Portuguese population [4], describing socio-cultural factors such as concern for health and finances as potential risk factors. Then, our results might also be considered in light of the existence of contextual differences during lockdown and socio-cultural aspects. As mood disturbances have been associated with a negative impact on eating symptoms among patients with ED [33], this study suggests whether those individuals with more adverse contextual conditions and a worse psychological state in the face of future similar adverse circumstances might be at greater risk of eating symptoms worsening in the middle and long term.

The scenario related to early pandemic and the restrictive measures adopted by governments differed between countries and could have contributed to the perceived emotional distress [2]. According to our results, the Brazilian individuals were those who had significantly higher percentages of people in charge, infection by COVID-19 and close people infected, in contrast with the other countries. This could be related to the fact that Brazilian patients were those who mostly kept working during the lockdown, with a presumably higher exposure to the infection added to the fact that social measures in the face of the pandemic appeared to be laxer. In this line, concerns related to both their own or their relatives’ health have been reported as potential stressors with a negative impact on mental health during the COVID-19 pandemic [22], as well as in previous health crises [23]. On the other hand, they also experienced higher financial losses during lockdown, which might be associated with a higher emotional distress.

In Brazil, as in other South American countries, aspects such as the lower social income of the population and a lack of infrastructures related to the public health system resulted in higher difficulties in access to treatment for mental health, including EDs. Despite all the participants of the study were already linked to specific treatment and aspects related to the evaluation of treatment during lockdown were not reported in this study, we hypothesized whether being subject to different socio-cultural and contextual conditions may have had an influenced in the adaptation and therapeutic adherence during this period and, therefore, in the perceived emotional distress. In this line, during the pandemic, most of the studies performed that reported the rapid implementation of telehealth care for EDs and other psychiatric conditions have been carried out in European, Australian, Asiatic, and North American countries so far [21, 40]. However, there is still a lack of information on the health policies of South American or African countries in this regard [24]. Then, both a regulation of the incomes and health care policies may be considered and improved in order to ensure that health care will be provided in those vulnerable populations despite the country of residence [24]. This fact becomes especially relevant when considering that poorer adaptive coping strategies to deal with emotional distress related to the pandemic and lockdown has been described as a factor of higher psychological vulnerability in patients with EDs [11–13]. Precisely, Brazilian participants showed lower resilience, which could also mediate the significant differences observed regarding greater emotional distress in this group.

This study has some limitations, such as a small simple size, an observational cross-sectional design, the lack of a control group, and the focus on the female clinical population already linked to a specific treatment unit, which could limit the generalization of the results. Besides, a voluntary participation, the retrospective collection of the data through a self-report way, and differences in the recruitment period between units are other limiting aspects, which could be associated with recall biases. On the other hand, some strengths should also be highlighted. For instance, the study contemplated potential co-founder factors in the analysis and the CIES is considered a validated and homogeneous psychometric instrument. However, future research is still needed to further investigate the clinical implications of mood disturbances related to pandemic situations on eating symptoms in the middle and long term, as well as potential meditational variables, such as sociodemographic and cultural factors.

In conclusion, the present study supports previous literature regarding the negative impact of the COVID-19 pandemic and lockdown on patients with EDs, adding a transcultural perspective with the inclusion of European and South American countries, and paying attention to the crucial role of mood disturbances and the sociodemographic context of the participants. Hence, more adverse contextual conditions, a worse psychological state, and poorer coping strategies may be potential contributing factors to the worsening of the eating symptoms in similar adverse situations.

## Supplementary Information

**Additional file 1**: **Table S1**. Descriptive for the age and the confinement context

**Additional file 2**: **Table S2**. Comparison of the post-pre differences by the ED-subtypes

**Additional file 3**: **Table S3**. Comparison of the post-pre differences by groups of age (adjusted by ED-subtype and country)

**Additional file 4**: **Table S4**. Comparison of the post-pre differences by country (adjusted by ED-subtype and age)

## Data Availability

The datasets generated and/or analysed during the current study are not publicly available to preserve participants’ privacy and due to there are ongoing studies using the data but are available from the corresponding author on reasonable request.

## Abbreviations

AN: Anorexia nervosa
ANOVA: Analysis of variance
BED: Binge eating disorder BED
BMI: Body mass index
BN: Bulimia nervosa
CFA: Factorial Analysis
CIES: COVID-19 isolation eating scale
CIES F1: CIES factor 1
CIES F2: CIES factor 2
CIES F3: CIES factor 3
CIES F4: CIES factor 4
CIES F5: CIES factor 5
DSM-5: Diagnostic and statistical manual of mental disorders, fifth edition
EDs: Eating disorders
ER: Emotion regulation
Emotion dysreg: Emotion dysregulation
OSFED: Other specified feeding and eating disorder
