# Supplementary material for: Eating disorders during lockdown: the transcultural influence on eating and mood disturbances in Ibero-Brazilian population
Source: J Eat Disord. 2023 Mar 11;11:39. doi: 10.1186/s40337-023-00762-7 (PMC10008014; doi:10.1186/s40337-023-00762-7)
Supplement: Supplementary file 1 — Additional file 1: Table S1. Descriptive for the age and the confinement context [file 40337_2023_762_MOESM1_ESM.docx]

**Table S1.** Descriptive for the age and the confinement context

|  | Portugal | | Brazil | | Spain | |  |
| --- | --- | --- | --- | --- | --- | --- | --- |
|  | *N=28* | | *N=111* | | *N=135* | |  |
| Age (yrs-old)*; mean - SD* | 29.39 | 8.87 | 39.18 | 12.91 | 30.08 | 11.35 | **.001*** |
| Contextual lock-down |  |  |  |  |  |  |  |
| Patient lived … Alone | 1 | 3.6% | 10 | 9.9% | 18 | 13.3% | .127 |
| With 1-2 people | 14 | 50.0% | 39 | 38.6% | 47 | 34.8% |  |
| With 3 people | 5 | 17.9% | 21 | 20.8% | 43 | 31.9% |  |
| With 4 or more people | 8 | 28.6% | 31 | 30.7% | 27 | 20.0% |  |
| Having care for… None | 16 | 57.1% | 41 | 40.6% | 114 | 84.4% | **.001*** |
| Someone | 12 | 42.9% | 60 | 59.4% | 21 | 15.6% |  |
| Patient infected No | 25 | 89.3% | 77 | 76.2% | 125 | 92.6% | **.001*** |
| Yes | 3 | 10.7% | 24 | 23.8% | 10 | 7.4% |  |
| Closed one infected No | 25 | 89.3% | 32 | 31.7% | 94 | 69.6% | **.001*** |
| Yes | 3 | 10.7% | 69 | 68.3% | 41 | 30.4% |  |
| Working No | 12 | 42.9% | 33 | 32.7% | 80 | 59.3% | **.001*** |
| Yes | 16 | 57.1% | 68 | 67.3% | 55 | 40.7% |  |
| Financial loses No | 18 | 64.3% | 45 | 44.6% | 94 | 69.6% | **.001*** |
| Yes | 10 | 35.7% | 56 | 55.4% | 41 | 30.4% |  |

***Note.*** SD: standard deviation.
