# Supplementary material for: Eating disorders during lockdown: the transcultural influence on eating and mood disturbances in Ibero-Brazilian population
Source: J Eat Disord. 2023 Mar 11;11:39. doi: 10.1186/s40337-023-00762-7 (PMC10008014; doi:10.1186/s40337-023-00762-7)
Supplement: Supplementary file 2 — Additional file 2: Table S2. Comparison of the post-pre differences by the ED-subtypes [file 40337_2023_762_MOESM2_ESM.docx]

**Table S2.** Comparison of the post-pre differences by the ED-subtypes

|  | AN | | BN | | BED | | OSFED | | *Significant* |
| --- | --- | --- | --- | --- | --- | --- | --- | --- | --- |
|  | *N=74* | | *N=44* | | *N=81* | | *N=65* | | *Pairwise* |
|  | *Mean* | *SD* | *Mean* | *SD* | *Mean* | *SD* | *Mean* | *SD* | *Comparisons* |
| Weight (kg) | -1.31 | 7.90 | 1.40 | 5.56 | 3.15 | 8.76 | -0.64 | 8.81 | (AN = OSFED) ≠ (BN=BED) |
| BMI (kg/m^2^) | -0.38 | 3.22 | 0.52 | 2.11 | 1.20 | 3.30 | -0.26 | 3.36 | (AN = OSFED) ≠ (BN=BED) |
| CIES-F1 ED symptoms | 0.53 | 4.98 | 1.78 | 6.21 | 0.79 | 4.42 | 0.35 | 5.46 | --- |
| CIES-F2 Eating style | -0.56 | 5.00 | 3.10 | 7.77 | -0.12 | 8.71 | 1.56 | 8.01 | (AN = BED) ≠ (BN=OSFED) |
| CIES-F3 Anxiety-dep. | 4.80 | 8.15 | 5.07 | 7.19 | 6.22 | 7.89 | 5.95 | 8.23 | --- |
| CIES-F4 Emot dysreg. | 1.35 | 3.24 | 1.43 | 2.81 | 1.31 | 2.63 | 1.40 | 4.06 | --- |

***Note.*** ED. Eating Disorder. BMI: body mass index. AN: anorexia nervosa. BN: bulimia nervosa. BED: binge eating disorder. OSFED: other specified feeding eating disorder. Anxiety-dep. Anxiety-depressive symptoms. Emot dysreg: Emotional dysregulation. SD: standard deviation. Results adjusted country and age.
