# Supplementary material for: Eating disorders during lockdown: the transcultural influence on eating and mood disturbances in Ibero-Brazilian population
Source: J Eat Disord. 2023 Mar 11;11:39. doi: 10.1186/s40337-023-00762-7 (PMC10008014; doi:10.1186/s40337-023-00762-7)
Supplement: Supplementary file 3 — Additional file 3: Table S3. Comparison of the post-pre differences by groups of age (adjusted by ED-subtype and country) [file 40337_2023_762_MOESM3_ESM.docx]

**Table S3.** Comparison of the post-pre differences by groups of age

|  | Younger age  *N=78* | | Older age  *N=186* | |  | |
| --- | --- | --- | --- | --- | --- | --- |
|  | *Mean* | *SD* | *Mean* | *SD* | *p* | *\|d\|* |
| Weight (kg) | -0.77 | 7.26 | 1.26 | 8.57 | .092 | 0.26 |
| BMI (kg/m^2^) | -0.30 | 2.72 | 0.51 | 3.33 | .081 | 0.27 |
| CIES-F1 ED symptoms | 1.74 | 5.96 | 0.49 | 4.76 | .106 | 0.23 |
| CIES-F2 Eating style | 0.90 | 7.00 | 1.05 | 7.80 | .895 | 0.02 |
| CIES-F3 Anxiety-depression symptoms | 6.57 | 8.46 | 5.07 | 7.66 | .206 | 0.19 |
| CIES-F4 Emotional dysregulation | 1.35 | 4.06 | 1.39 | 2.80 | .936 | 0.01 |

***Note.*** ED. Eating Disorder. BMI: body mass index. SD: standard deviation. *Bold: significant comparison. ^†^Bold: Effect size into the ranges moderate to large. Results adjusted by ED-subtype and country.
