# Supplementary material for: Eating disorders during lockdown: the transcultural influence on eating and mood disturbances in Ibero-Brazilian population
Source: J Eat Disord. 2023 Mar 11;11:39. doi: 10.1186/s40337-023-00762-7 (PMC10008014; doi:10.1186/s40337-023-00762-7)
Supplement: Supplementary file 4 — Additional file 4: Table S4. Comparison of the post-pre differences by country (adjusted by ED-subtype and age) [file 40337_2023_762_MOESM4_ESM.docx]

**Table S4.** Comparison of the post-pre differences by country

|  | Portugal | | Brazil | | Spain | | Portugal | | Portugal | | Brazil | |
| --- | --- | --- | --- | --- | --- | --- | --- | --- | --- | --- | --- | --- |
|  | *N=28* | | *N=101* | | *N=135* | | vs Brazil | | vs Spain | | vs Spain | |
|  | *Mean* | *SD* | *Mean* | *SD* | *Mean* | *SD* | *p* | *\|d\|* | *p* | *\|d\|* | *p* | *\|d\|* |
| Weight (kg) | 1.37 | 5.07 | 1.64 | 9.78 | -0.21 | 7.40 | .882 | 0.03 | .349 | 0.25 | .132 | 0.21 |
| BMI (kg/m^2^) | 0.51 | 1.95 | 0.65 | 3.65 | -0.05 | 2.97 | .840 | 0.05 | .394 | 0.22 | .141 | 0.21 |
| CIES-F1 ED symptoms | 0.19 | 5.01 | 1.26 | 5.25 | 0.73 | 5.16 | .360 | 0.21 | .615 | 0.11 | .505 | 0.10 |
| CIES-F2 Eating style | 0.40 | 5.70 | 2.39 | 9.03 | 0.15 | 6.63 | .239 | 0.26 | .873 | 0.04 | **.050*** | 0.28 |
| CIES-F3 Anxiety-depress. | 2.31 | 4.95 | 7.78 | 8.00 | 4.63 | 8.08 | **.002^†^** | **0.82*** | .156 | 0.35 | **.008*** | 0.39 |
| CIES-F4 Emotional dysreg. | 0.20 | 1.67 | 1.79 | 2.89 | 1.34 | 3.63 | **.029^†^** | **0.67*** | .091 | 0.40 | .354 | 0.14 |

***Note.*** ED. Eating Disorder. BMI: body mass index. Anxiety-depress.: Anxiety-depressive symptoms. Emotional dysreg.: Emotional dysregulation. SD: standard deviation. *Bold: significant comparison. †Bold: Effect size into the ranges moderate to large. Results adjusted by ED-subtype and age.
